# Isolated specific IgA against respiratory viruses, Influenza or SARS-CoV-2, present in the saliva of a fraction of healthy and asymptomatic volunteers

**DOI:** 10.1016/j.clinsp.2022.100105

**Published:** 2022-09-06

**Authors:** Nahiara Esteves Zorgi, Luciana R. Meireles, Danielle Bruna Leal Oliveira, Danielle Bastos Araujo, Edson L. Durigon, Heitor Franco de Andrade Junior

**Affiliations:** a Faculdade de Medicina da Universidade São Paulo (FMUSP), São Paulo, SP, Brazil; b Laboratório de Protozoologia, Instituto de Medicina Tropical de São Paulo (IMTSP), São Paulo, SP, Brazil; c Instituto de Ciências Biomédicas, Universidade de São Paulo (USP), São Paulo, SP, Brazil

**Keywords:** Influenza, SARS-CoV-2, IgA, IgG, Saliva

## Abstract

**Objectives:**

Defense against respiratory viruses depends on an immune response present in the mucosa, as saliva IgA secretes antibodies. During the pandemic, such as influenza or SARS-CoV-2, most infected patients are asymptomatic but retain specific antibodies post-infection. The authors evaluated IgG and IgA antibodies against SARS-CoV-2 and influenza in the saliva of asymptomatic volunteers, validated with controls or vaccinated individuals.

**Methods:**

The authors detected specific antibodies by validated conventional ELISA using natural SARS-CoV-2 antigens from infected Vero cells or capture-ELISA for influenza using natural antigens of the influenza vaccine.

**Results:**

Saliva from influenza-vaccinated individuals had more IgA than paired serum, contrary to the findings for specific IgG. In COVID-19-vaccinated samples, specific IgA in saliva increased after vaccination, but IgG levels were high after the first dose. In saliva from the asymptomatic population (226), *anti-Influenza* IgG was found in 57.5% (130) of samples, higher than IgA, found in 35% (79) of samples. IgA results were similar for SARS-CoV-2, with IgA present in 30% (68) of samples, while IgG was less present, in 44.2% (100) of samples. The proportion of influenza IgG responders was higher than that for SARS-CoV-2 IgG, but both populations presented similar proportions of IgA responders, possibly due to variable memory B cell survival. For both viruses, the authors found an important proportion (> 10%) of IgA+IgG- samples, suggesting the occurrence of humoral immunity directed to the mucosa.

**Conclusion:**

Specific antibodies for respiratory viruses in saliva are found in either infection or vaccination and are a convenient and sensitive diagnostic tool for host immune response.

## Highlights

• Natural antigen ELISA detected saliva IgG or IgA against respiratory viruses in asymptomatic people from Influenza and COVID.
• Samples with specific influenza IgG were more frequent than COVID IgG, probably due to long time exposure to influenza, but samples positive for specific IgA had same proportion for both viruses.

## Introduction

Pandemic respiratory viruses cause morbidity and mortality worldwide, either as asymptomatic spreaders or lethal Severe Acute Respiratory Syndrome (SARS). This disease pattern is caused by a variety of viruses, including influenza and coronavirus.^1^ Coronavirus Disease 2019 (COVID-19) caused by Severe Acute Respiratory Syndrome due to Coronavirus 2 (SARS-CoV-2) emerged in China in late 2019, with cases of severe pneumonia and diffuse alveolar damage. Since then, SARS-CoV-2 infections have been widespread worldwide and have possibly caused the most serious pandemic since the Spanish flu.^2^^,^^3^ Influenza is also prevalent worldwide, and approximately 10% of the world population experiences an episode of influenza annually. Seasonal influenza epidemics are estimated to result in 3‒5 million cases of serious illnesses with approximately 290,000‒650,000 deaths from respiratory causes worldwide.^4^

Influenza virus vaccines induce short-lasting humoral and cell-mediated immunity and must be administered yearly for partial protection.^5^ CD4+ T-helper cells help the immune response to influenza in multiple ways, including memory as well as the differentiation of “naïve” B-cells into IgA secreting plasma cells, mainly secretory IgA (sIgA).^6^ sIgA antibodies are the main antibody isotypes present in external secretions, such as nasal fluid, saliva, milk, intestinal colostrum, and gallbladder bile.^7^ Independent of TCD4 cells, Antigen Presenting Cells (APC) activation is essential for the development of strong responses of effector TCD8 cells, leading to long-term protective memory.^8^ The humoral response is able to neutralize the virus and thus prevent invasion into the target cells, and the cell-mediated immune response is able to prevent influenza virus replication in infected cells, consequently decreasing the patient's recovery time.^9^^,^^10^

The transition between innate and adaptive immune responses appears essential for SARS-CoV-2 infection, and unknown immunological regulatory events are associated either with the development of a protective immune response or an exacerbated inflammatory response.^11^ TCD4 cells specific for SARS-CoV-2 help B-cells produce specific neutralizing antibodies and activate cytotoxic TCD8 cells capable of eliminating infected cells.^12^

Efforts to prevent the impact of pandemics of both seasonal influenza and SARS-CoV-2 have focused on the use of vaccines.^13^^,^^14^ Influenza virus, with a high mutation ratio and multiple hosts, often causes epidemics with new variants, to which the population has no immunity; influenza also regularly infects patients who have lost, or are incapable of producing effective immunity.^15^ For COVID-19, a relatively small number of mutations can mediate the inactivity of the vaccine.^16^

During the current pandemic, vaccines against SARS-CoV-2 that induce protective immune responses are crucial for the prevention and reduction of morbidity and mortality. Studies have indicated that a balanced response of humoral and cellular immunity directed by a Th1-type immune response may be important for protection against COVID-19. Several candidate vaccines are being developed and tested, including nucleic acid vaccines, inactivated viruses, live attenuated viruses, subunit or viral vector proteins, and peptides.^17^ For this study, the available vaccine authorized for emergency use was CoronaVac® (Sinovac Biotech), a chemically inactivated vaccine that was initially produced in China and used in the country in July 2020.

Currently available vaccines for influenza and COVID-19 are parenterally injected intramuscularly, inducing a systemic immune response and not directly a mucosal immune response. The sIgA antibodies present in the mucosa of the upper part of the respiratory tract, together with innate immunity and natural mucosal barriers, are essential to prevent infection of the respiratory tract and transmission of the virus.^18^ Investigating the presence of IgA in the secretions of patients with infections or those vaccinated for influenza or COVID-19 is of great importance to define possible neutralizing antiviral activities in the respiratory tract mucosa. Here, the authors analyzed the presence of specific IgG and IgA antibodies in saliva for influenza or COVID-19 in asymptomatic adult volunteers from São Paulo in assays validated with samples from influenza and COVID-19 vaccines, to understand the specific humoral response in respiratory viral infections.

## Material and methods

### Sample acquisition

The authors retrieved two groups of serum samples with known serology from the laboratory biorepository to validate saliva assays. The first positive group included samples from people vaccinated with influenza or those enrolled in the CoronaVac vaccine study, collected before or ten days after each vaccine dose. The second group, negative serum samples, was composed of pools of four serum samples collected from 6‒9 months old infants for routine diagnostic laboratory tests before the pandemics from a repository of previous work on vaccine controls.^19^ These samples were negative for most respiratory virus infections and were devoid of maternal protective antibodies.

Asymptomatic volunteers, mainly students or employees from universities, hospitals, and associated companies, were invited to participate in the study at several companies in the São Paulo metropolitan area. All the volunteers were adequately informed of the scope of the study and voluntarily signed an informed consent form from August 2020 to March 2021. The volunteers provided only two self-collected saliva samples in the Salivette® system (SARSTEDT), either directly or after cleansing the mouth. There were no differences in sex proportions, with 116 women and 110 men in the sample. The mean age was also similar, with 41.4 years (Standard Deviation, SD 13.3 years) for women and 39.6 years (SD 15.8 years) for men. The local and Brazilian Research Ethics Committee (Plataforma Brasil nº 34742520.0.0000.0068) approved this study.

### Preparation of saliva

After collection, the device was centrifuged at 4,000 rpm at 4°C for 5 min to recover clean saliva, the intermediary from the upper reservoir of tampon, and the pellet reservoir. Saliva (1 mL) was added to tubes containing chilled absolute Ethanol (1 mL), vortexed, and stored overnight at -20°C. The precipitated antibodies and globulins were pelleted by centrifugation at 10,000 rpm for 5 min. The pellet was suspended in 400 µL of saline borate buffer Ph 7.2 with 1 mg/mL of BSA (Serum Bovine Albumin, Sigma®), resulting in 2.5× concentrated saliva, which was then stored at -20°C until use.

### Immunoenzymatic assay for the detection of specific IgG and IgA anti-SARS CoV- 2 antibodies

Influenza antigens were recovered from unused and discarded past year's commercial influenza vaccines kindly provided by the Immunization Center of Hospital das Clínicas da Faculdade de Medicina da Universidade de São Paulo. The vaccine proteins were purified from the diluent and interfering small molecules by chromatography on a molecular exclusion column in Sephadex G-25 in 0.1 M sodium carbonate buffer (pH 9.0). Purified antigen fractions > 5 KDa were associated with biotin ligands via NHS, using a fabricant protocol (EZ-Link NHS-LC-Biotin, Thermo Scientific࣪), resulting in a final molar ratio of four biotins per 30 kDa molecules. Natural SARS-CoV-2 antigens from the infected Vero monolayers were prepared and stored as previously described.^20^ The detection of IgG- and IgA-specific antibodies in the serum and saliva of volunteers was carried out using capture-ELISA for influenza or conventional ELISA for SARS-CoV2. For the influenza capture-ELISA, the plates were initially sensitized with *Staphylococcus aureus* protein A (1 µg/mL) (Sigma®) to capture IgG or *anti-human* IgA (Sigma®) (1 µg/mL). For the conventional SARS-CoV-2 ELISA, 96-well high-affinity polystyrene plates were coated with natural SARS-CoV-2 antigens from VERO cell infections at 10 µg/mL overnight. The remaining binding sites were blocked with PBS containing 0.05% Tween-20 (PBST) and 1% skim milk (Molico®) for 1h at 37°C. During all the subsequent steps, the wells were washed five times with PBST. The saliva (2.5×) or serum samples (1/500) adequately diluted in PBST were incubated for 1 h at 37°C for both assays.

The proportion of antibodies specific to influenza was determined by the addition of biotinylated viral vaccine antigens for 1h at 37°C, revealed after washing with avidin peroxidase complex (Sigma-Aldrich®). For SARS-CoV-2 IgG and IgA, the bound antibody was removed by incubation with anti-human IgG or human IgA peroxidase conjugates. Peroxidase content was determined by the addition of 100 µL/well of TMB (3.3′, 5.5′ ‒ tetramethylbenzidine dihydrochloride) (Sigma-Aldrich®) for 30 min and interrupted by the addition of 50 µL of H_2_SO_4_ 2M. The absorbance of each well was determined using a microplate spectrophotometer at 450 nm. Using these controlled samples, the authors calculated the serological indices of the study's assays for both IgG and IgA for each respiratory virus, as shown in Table 1. As could be seen, the assays were validated as highly sensitive and specific, usually over 95% for most indexes, viruses, and immunoglobulins. SARS CoV2 IgG specificity was near 95%, which could be due to first dose-vaccinated low responders.

**Table 1:** Serological indexes of the assays in the present study's samples different systems, using commercial assays defined samples.

	Influenza		SARS-CoV 2	
	IgG	IgA	IgG	IgA
**Positive samples**	34	34	28	28
**Negative samples**	29	29	28	28
**Sensitivity (95% CI)**	0.967 (0.828 to 0.999)	1 (0.880 to 1)	1 (0.868 to 1)	0.966 (0.822 to 0.999)
**Specificity (95% CI)**	1 (0.894 to 1)	1 (0.897 to 1)	0.933 (0.779 to 0.992)	1 (0.872 to 1)
**Positive predictive value (95% CI)**	1 (0.881 to 1)	1 (0.881 to 1	0.929 (0.765 to 0.991)	1 (0.877 to 1)
**Negative predictive value (95% CI)**	0.971 (0.847 to 0.999)	1 (0.897 to 1)	1 (0.877 to 1)	0.964 (0.816 to 0.999)

### Statistical analysis

All statistical analyses used individual data in Artificial Units. Artificial Units were the ratio of Optical Density (O.D.) 450 nm in samples and the upper 95% Confidence Interval of O.D. from negative control samples in each assay. The authors performed a comparison between quantitative values by Analysis of Variance (ANOVA), using all groups together, with homogeneity of variances checks and Bonferroni multiple comparison post-test. Comparisons were considered significant when the probability of equality was less than 5% (*p* < 0.05). All statistical calculations were performed using GraphPad Prism 7.0.

## Results

### Influenza serology in vaccinated controls

**Fig. 1:**
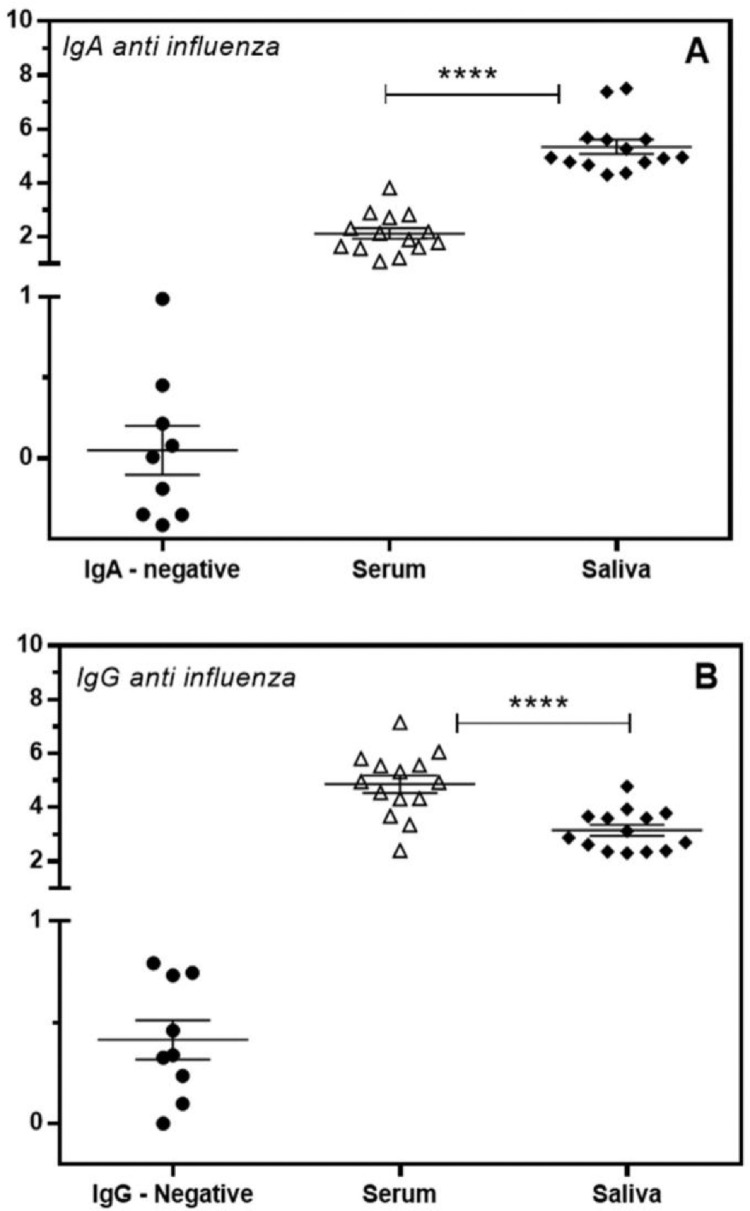
Detection of IgA (A) and IgG (B) antibodies specific for influenza in the serum (open triangle) and saliva (closed diamond) of vaccinated volunteers. Individual values are expressed as Artificial Units. Significant differences are marked by asterisks between the indicated groups (****p < 0.0001), determined by Bonferroni posttests.

The authors analyzed the specific humoral immune response in paired serum and saliva samples of healthy 2020 influenza-vaccinated individuals (*n* = 14). The detection of specific antibodies was carried out by immunoenzymatic capture assays using protein A and *anti-human IgA* to capture IgG and IgA from biological samples, respectively, with specificity revealed by biotin-labeled influenza virus natural antigens. Vaccination for influenza showed a specific humoral response both at the systemic and mucosal levels, with the production of *anti-influenza* IgG and IgA. The production of specific IgA antibodies was significantly different between serum and saliva, showing a higher proportion of IgA levels in saliva, whereas the authors observed significantly higher proportions of specific IgG in serum (Fig. 1). These assays were highly efficient in either source, serum, or saliva, without false-negative or false-positive results.

### SARS-CoV-2 serology in vaccinated controls

**Fig. 2:**
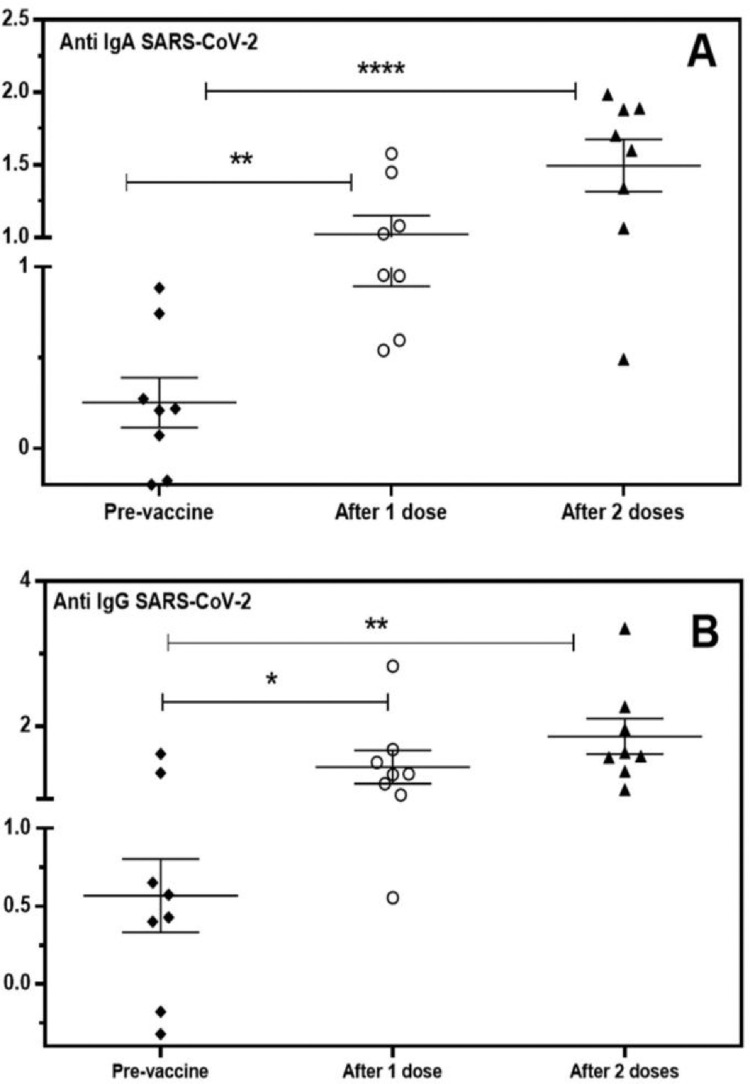
Detection of specific IgA (A) and IgG (B) antibodies for SARS-CoV-2 in the saliva of volunteers vaccinated with CoronaVac®. Individual values are expressed as Artificial Units. Significant differences are marked by asterisks between the indicated groups (*p < 0.05, **p < 0.01, ****p < 0.0001), determined by Bonferroni posttests.

Concurrently with the emergency vaccination for COVID-19 using CoronaVac® (Institute Butantã, SP, Brazil) of the health personnel, the authors evaluated the specific humoral immune response in the saliva of healthy individuals and health workers (*n* = 8) after one or two doses of the immunizer. The authors used conventional immune enzymatic assays with total natural antigens of SARS-CoV-2 virions from *in vitro* infected Vero cells, which contain mainly nucleoprotein and spike proteins, similar to the type of vaccine used.^20^ The authors detected IgA and IgG antibodies in saliva before the vaccine (pre-vaccine), 15 days after the 1^st^ dose (after 1 dose), and 15 days after the 2^nd^ vaccine dose (2 doses). As shown in Fig. 2, two individuals were positive for *anti-SARS-CoV-2* IgG before the 1^st^ dose (pre-vaccine), possibly because of natural infection before vaccination. Fifteen days after the first dose and after the second dose, most individuals responded adequately with high IgG levels in saliva, as expected in a parenteral vaccination (Fig. 2B). Specific *anti-SARS-CoV-2* IgA was present after the first dose in 50% of the samples (4/8); however, after the second immunization, most patients presented a higher response (7/8) in salivary IgA (Fig. 2A). Both responses were highly significant in relation to the antibody levels before the immunizer. These data clearly demonstrated the efficiency of the serological assays for COVID-19 specific antibody detections.

### Detection of IgG and IgA antibodies, anti-Influenza and anti-SARS-CoV-2, in asymptomatic volunteers

**Fig. 3:**
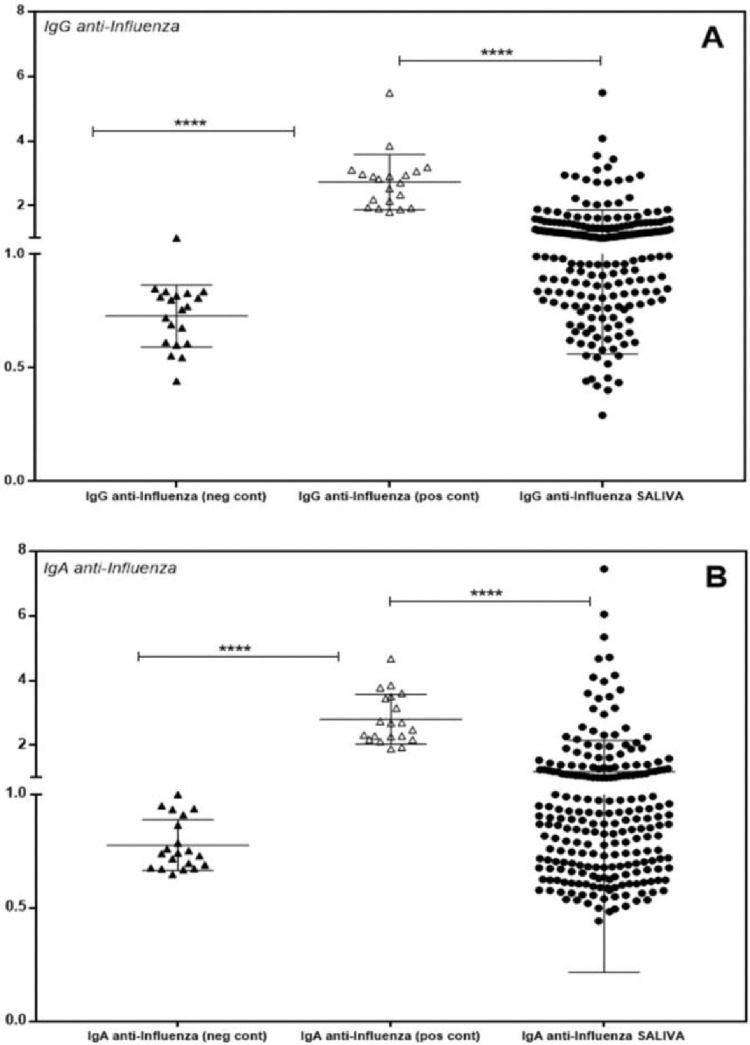
Individual detection of IgG (A) and IgA (B) antibodies specific for influenza in the saliva of volunteers. Individual values are expressed as Artificial Units. Significant differences are marked by asterisks between the indicated groups (****p < 0.0001), determined by Bonferroni posttests.

**Fig. 4:**
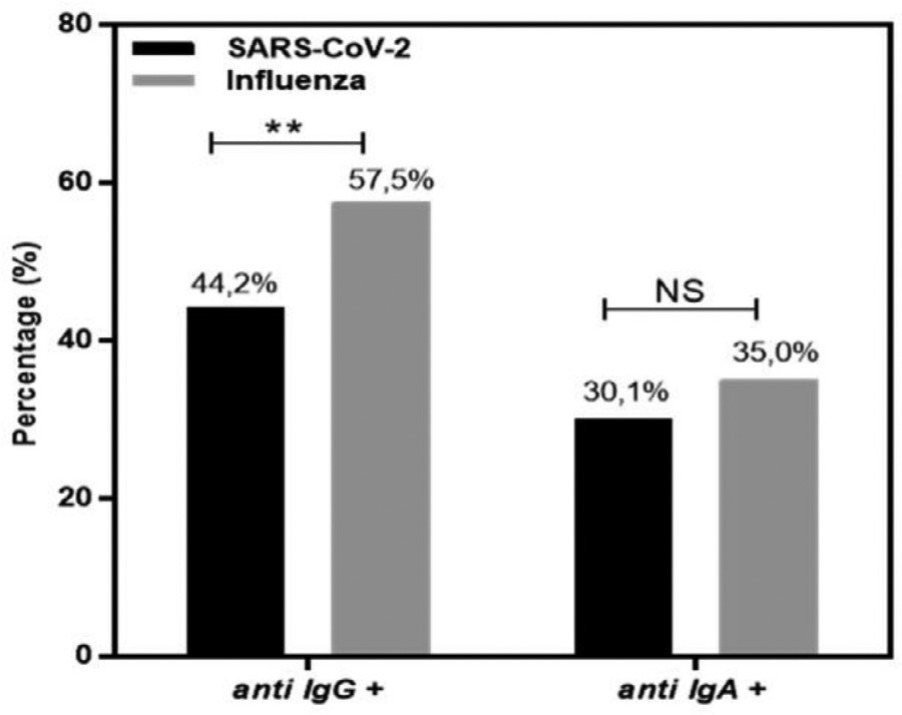
Percentage of individuals positive for SARS-CoV-2 (black bar) and influenza (gray bar) specific IgG and IgA. Significant differences are marked by asterisks between the indicated groups (**p < 0.01), determined by Chi square tests. NS, not significant.

The authors evaluated 226 saliva samples from healthy and asymptomatic individuals (*n* = 226) for specific influenza or SARS-CoV-2 IgG and IgA antibodies. None of the volunteers were COVID vaccinated or presented with any symptoms of COVID-19 or respiratory viral infection. The authors invited adults to participate, and saliva was collected before the influenza vaccination campaign, between August 2020 and February 2021. The study population was heterogeneous, including individuals from the health area, university students, and private company workers, aged 18 to 74 years, with similar gender proportions and mean age. In Fig. 3, the authors observe the dispersion of individuals positive for specific IgG and IgA in the saliva for influenza, compared with the negative control infant population and positive vaccinated controls. Negative and positive controls were confirmed by conventional serology in an externally certified laboratory. The present data showed that 57.5% (130/226) were positive for *anti-Influenza* IgG and 35.0% (79/226) for *anti-Influenza* IgA (Fig. 4).

**Fig. 5:**
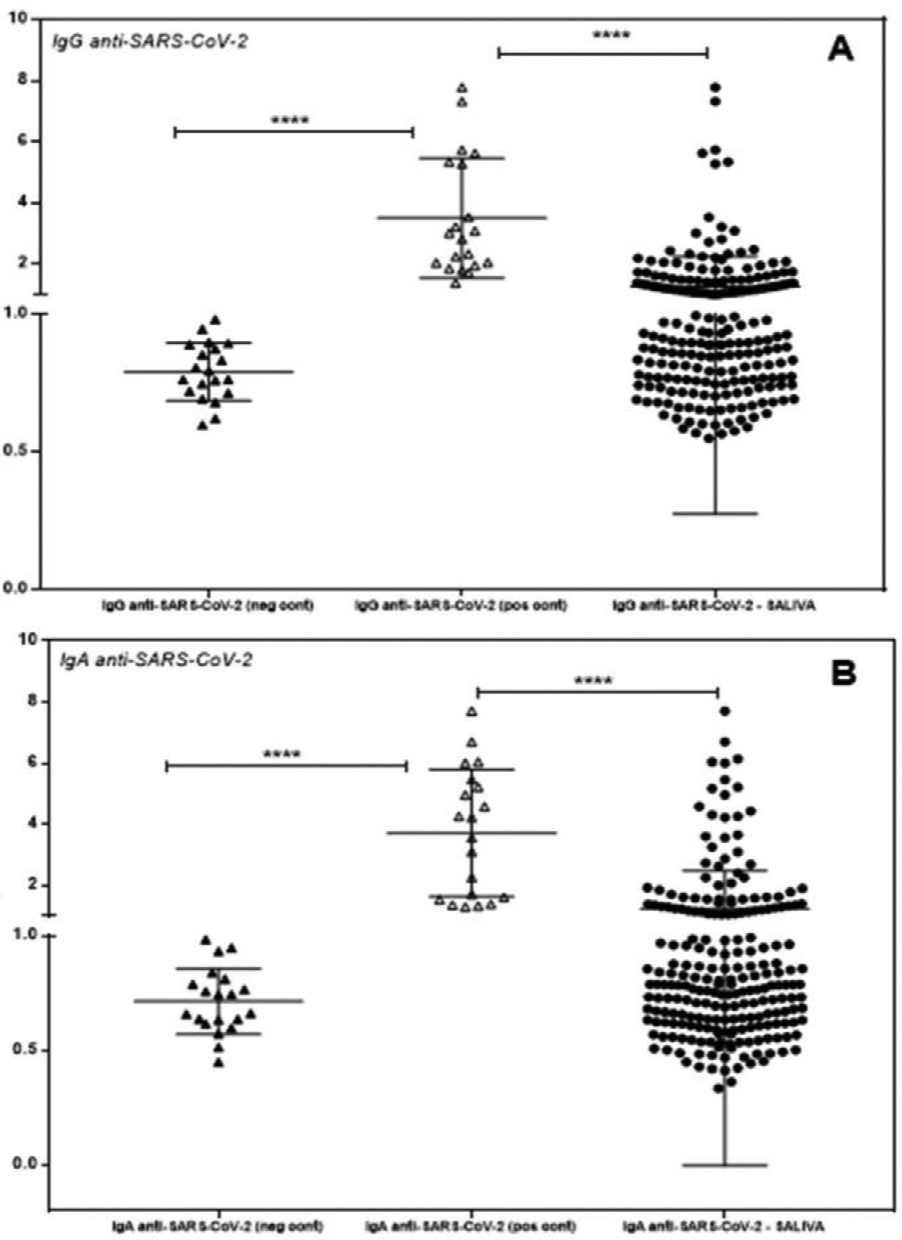
Individual detection of specific IgG (A) and IgA (B) antibodies for SARS-CoV-2 in the saliva of volunteers. Individual values are expressed as Artificial Units. Significant differences are marked by asterisks between the indicated groups (****p < 0.0001), determined by Bonferroni posttests.

In Fig. 5, the authors observe the dispersion of individuals positive for specific IgG and IgA in the saliva for SARS-CoV-2, compared with the negative infant and positive serology vaccinated populations. The authors found that 44.2% (100/226) were positive for *anti-SARS-CoV-2* IgG and 30.1% (68/226) for *anti-SARS-CoV-2* IgA (Fig. 4).

### Comparison of proportions of IgG and IgA positive samples in asymptomatic volunteers

The isolated proportion of asymptomatic samples positive for antibodies may represent the population exposed to and infected by a respiratory virus. As shown in Fig. 4, the authors compared the percentage of samples positive for IgG, with a higher proportion of positive samples for influenza (*p* > 0.01) than SARS-CoV2, showing that influenza, has been prevalent in recent years or presents a residual vaccine response. IgA antibodies have a short half-life, and as expected, the proportion of positive samples was similar among viral infections.

**Fig. 6:**
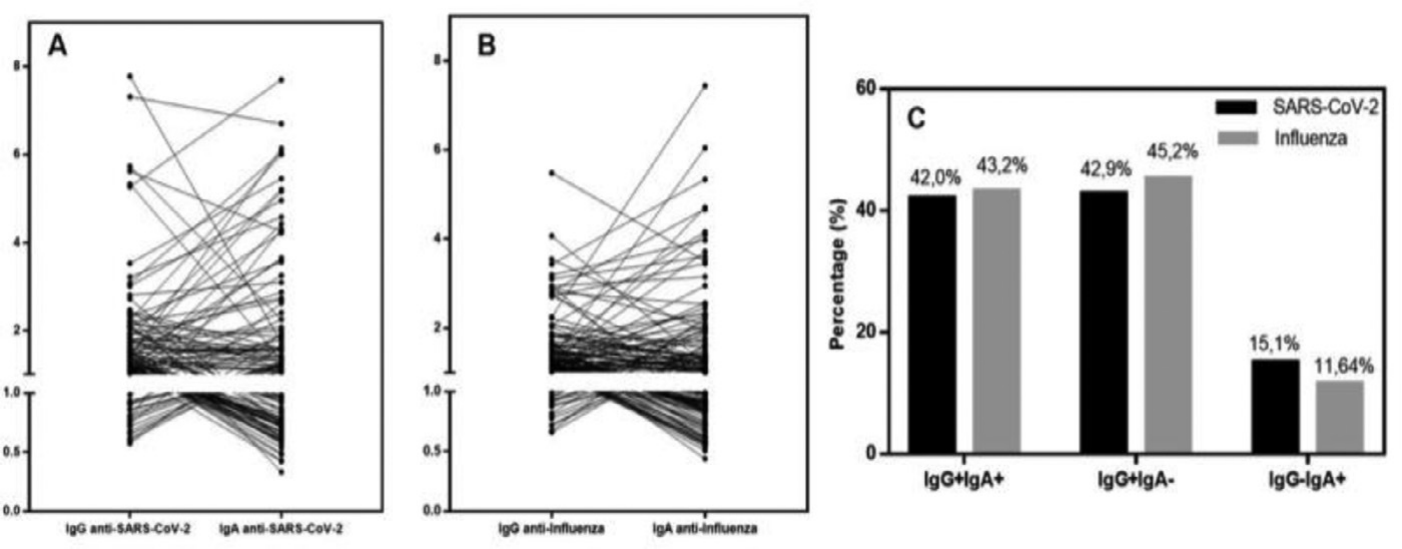
Correlation between specific IgG and IgA for SARS-CoV-2 (A) and influenza (B) in the group of positive individuals. Individuals’ values are expressed as Artificial Units. (C) Percentage of IgG+IgA+, IgG+IgA-, and IgG-IgA+ for SARS-CoV-2 (black bar) and influenza (gray bar) in exposed infected individuals, excluding negative samples. All proportions are similar when restricted to any immunoglobulin positive population.

Sorting for both antibodies in the studied population and excluding non-reactive antibodies, the authors found three positive groups: specific antibodies IgG+IgA+, IgG+IgA-, and IgG-IgA+ for influenza or COVID19; Fig. 6 shows all the effects. The authors first show the individual correlation of specific IgG and IgA for SARS-CoV2 (Fig. 6A) and influenza (Fig. 6B); all individual patterns can be seen. Some curves are highly ascending for IgA production, showing a mucosal response, while most samples are low-responsive IgG responders, without significant IgA in both infections. The authors analyzed the proportion of response of sorted groups in exposed infected samples, excluding samples without any positive result and considering antibody class production in three groups, which could represent a specific humoral response based on the half-life of those classes: IgG+IgA- sample representing residual immunity, IgG+IgA+ representing active immunity, and IgG-IgA+ representing a mucosal selective response. Fig. 6C shows a comparison of the proportions of responsive individuals, and the authors observed that there was no significant difference between the proportions of sorted groups of specific IgG+IgA+, IgG+IgA-, and IgG-IgA+ for influenza and SARS-CoV-2, showing the same general pattern of antibody production and secretion in the immune response to respiratory virus infection.

## Discussion

The present study's assays for antibody detection against respiratory viruses in saliva were effective and reliable despite the presence of very low amounts of antibodies in highly diluted serum samples or concentrated saliva. In capture assays, the capture agent in the solid support segregates the same amount of whole antibodies, avoiding the effect of varying saliva concentration, resulting in the detection of the proportion of specific antibodies, which is similar but not the same as the quantitated bound antibody in ELISA. The use of natural antigens improves the reliability of assays, and antigens, such as influenza vaccines produced by egg infections or shed viruses from cell cultures for SARS-CoV2, were from natural occurring viruses rather than recombinant bacterial proteins. In fact, the used systems are gold standards, as they use antigens from viable viruses as used for vaccine formulation,^21^ before the generalization of the use of bacterial recombinant antigens, with refolding or posttranslational processing problems.^22^

The authors clearly demonstrated the presence of specific IgG and IgA in the saliva of asymptomatic, unvaccinated volunteers. Antibodies produced by activated B-cells play a key role in antiviral immunity through several mechanisms including viral neutralization, cell cytotoxicity, cell phagocytosis, and complement activation.^23^ The generation of high levels of neutralizing antibodies for both influenza and SARS-CoV-2 is necessary for successful human vaccination. Influenza and SARS-CoV-2 are similar in terms of methods and modes of transmission, clinical characteristics, seasonal coincidence, and immune response.^24^ Currently, influenza vaccination is the most efficient, valuable, and low-cost tool to reduce influenza virus infection and its associated morbidity and mortality.^25^ In the case of COVID-19, mass vaccination of the world's population would be ideal for controlling the pandemic.

Conventional inactivated vaccines against influenza are administered parenterally by subcutaneous or muscle inoculation of the immunogen, activating the systemic immune response with increased levels of serum antibodies, especially IgG. However, few studies have evaluated this response to the vaccine in a mucosal system, such as the detection of antibodies in saliva, and usually adapting commercial serum tests.^26^ Some authors argue that systemic immunization is not able to generate an immune response in the mucosa with the secretion of antibodies; this response would only be possible with immunization via the mucosa.^27^ However, the present data showed that 57.5% had IgG antibodies and 35.0% had *anti-Influenza* IgA in the saliva of a population in São Paulo. Most of the vaccinated samples also presented both IgA and IgG in saliva; thus, systemic vaccination induces mucosal secretion of IgA.

The adult population had been exposed in the past to influenza but not to SARS-CoV-2, a recent exposure in the last year. This differential exposure explained the difference between IgG and IgA anti-influenza prevalence, but it was not present for IgG and IgA ant- COVID prevalence. Both viruses usually present only infection restricted to the respiratory epithelial cells exposed to saliva, the place of the first encounter. Despite the highly sensitive technologies used, most studies focus only on specific IgG detection in saliva^26^ which is not secreted into saliva but leaks from interdental crevicular fluid, increasing the risk of false-negative samples, as shown in reported assays.^28^ The present study's capture assays overcame these problems by testing a fixed amount of captured immunoglobulin from any source and avoiding the problem of varying concentrations in the saliva.

The authors detected a significant group of positive individuals who had only specific IgA antibodies and possibly undetectable IgG in their saliva. This group would go unnoticed by conventional IgG tests, representing a protected group that would be undiagnosed using only IgG detection in serum. SARS-CoV2 ELISA corroborates their occurrence because several patients had higher IgA levels in saliva with negative IgG. This was also confirmed for influenza samples, which showed isolated saliva IgA responders by more reliable saliva antibody capture assays.^19^ The authors can speculate that for respiratory viruses, the early production of specific IgA by memory cells could protect against infection, and this early control diminishes the activation of systemic IgG memory cells as IgA is the earliest neutralizing antibody produced in those infections,^29^ resulting in asymptomatic infections.

Passive transfer of IgA *anti-influenza* antibodies in naïve experimental models plays a protective role in experimental mice models.^30^ Human volunteer influenza vaccinees responded with the production of specific IgG antibodies in the serum and IgA in the mucosa, and the IgAs of origin of the nasal wash, but not serum IgG, were responsible for the neutralization activity.^31^ Secretory dimeric IgA may have a greater capacity to neutralize viral particles than monomeric IgA present in serum.^32^

Multiple evidence in SARS-CoV-2 support that the humoral response, main antibodies against protein S, blocks the virus from binding to susceptible cells.^33^ However, there are still many questions regarding the significance of antibodies against different viral proteins and the cross-reactivity of antibodies against other highly prevalent alpha and beta-coronaviruses.^34^ IgM and IgA antibodies can be detected in the first week of symptom onset, whereas IgG can be detected approximately 14 days after;^35^ however, it is not known for how long the levels of protection of these blocking antibodies will remain active. Several studies on COVID-19 have shown the presence of serum IgA against SARS-CoV-2^35^ and, in preclinical studies with anti-SARS vaccines administered sublingually or intranasally, neutralizing IgA in bronchoalveolar washings.^36^^,^^37^ Only a few reports have dealt with salivary IgA in the recent SARS-Cov2 pandemic, instead focusing on clinically symptomatic nasal RNA+ acute patients who are acutely infected and have low protection.^38^ Most other studies used commercial assays for IgA detection, which were prepared for serum and had problems when testing saliva.^39^ These findings support the importance of investigating the presence of specific IgA in the secretions of patients with COVID-19 due to the possible neutralizing antiviral activity in the mucosa of the respiratory tract.^29^

## Conclusions

The authors studied the humoral response against SARS-CoV-2 and influenza in saliva, an important tool for vaccine design and comprehension of the repertory viral immune response. It appears that the IgA response must be rapid, intense, and selective for mucosal protection against invasion, but this protection is costly to the host, resulting in similar rapid control in the absence of aggression. The present study's data obtained from asymptomatic volunteers showed a significant group of infected people without detectable IgG, but easily determined specific saliva IgA. Mucosa-restricted immunity could occur, and studies with only serum could fail to understand the existing protection in a sample or population. In summary, the present results indicate that the detection of specific IgG and IgA antibodies in the saliva is easy, possible, useful, and a valuable tool for the diagnosis of respiratory viral infections and vaccine evaluation.

## Funding

This project was funded by CAPES (Coordenação de Aperfeiçoamento de Pessoal de Nivel Superior; proc. nº 88881.507232/2020-01) a Brazilian government funding agency. We gratefully thank the cooperation of asymptomatic volunteers that provide saliva samples for our assays.

## Conflicts of interest

The authors declare no conflicts of interest.
